# Relationship Between Semen IL-6, IL-33 and Malondialdehyde Generation in Human Seminal Plasma and Spermatozoa

**DOI:** 10.1007/s43032-021-00493-7

**Published:** 2021-02-23

**Authors:** Elena Moretti, Daniela Cerretani, Daria Noto, Cinzia Signorini, Francesca Iacoponi, Giulia Collodel

**Affiliations:** 1 Department of Molecular and Developmental Medicine, University of Siena, 53100 Siena, Italy; 2 Department of Medical and Surgical Sciences and Neurosciences, University of Siena, 53100 Siena, Italy; 3 Department of Food Safety and Veterinary Public Health, Istituto Superiore di Sanità, Viale Regina Elena, 299, 00161 Rome, Italy

**Keywords:** Human semen, IL-6, IL-33, MDA, Spermatozoa

## Abstract

Cytokines are physiological seminal components and their abnormal levels, reported in different pathological conditions, negatively influence the sperm function. We analysed the relationship between interleukin (IL)-6 and IL-33 levels and lipid peroxidation (LPO), measured both in semen and sperm lysate, in 44 human semen samples. The semen analysis was performed following the WHO guidelines. Seminal IL-6 and IL-33 concentrations were assessed by ELISA and LPO was evaluated measuring malondialdehyde (MDA) both in seminal plasma and viable spermatozoa. Two small groups of patients with varicocele and infection were extrapolated from the cases analysed and the variables compared with those of a group of control. IL-33 levels were undetectable in all samples and IL-6 levels were positively correlated with both seminal and sperm MDA concentrations (*p* < 0.01) and negatively with sperm parameters (*p* < 0.01). Seminal and sperm MDA levels were both negatively correlated with sperm parameters (*p* < 0.01). IL-6 and semen MDA showed an exponential positive relationship, whereas MDA values measured in viable spermatozoa were low until IL-6 amount reached a concentration of >30 pg/mL, rising consistently. By comparing the variables in the groups, we confirmed that a high IL-6 concentration in the varicocele and infection groups was concomitant with an increase of seminal MDA levels, but also with MDA measured in viable spermatozoa, which represents the novelty of this study. We identified the IL-6 threshold, beyond which sperm MDA concentration rises concomitantly with the increase of IL-6 concentration. Other studies are needed, considering the increasing number of patients with different pathologies affecting male infertility.

## Introduction

Proinflammatory cytokines are mediators produced and secreted by both cells of the immune system and cells that are part of tissues, in response to external stimuli and other cytokines. They are the main actors of inflammatory reactions and can be considered regulatory factors in different biological processes [1]. Seminal plasma contains several cytokines, which are physiologically present in the male genital tract. These cytokines can be released by Leydig cells, Sertoli cells, the epididymis and prostate, and their expression is regulated during the seminiferous epithelium cycle [2]. For example, interleukin (IL)-6 affects spermatogonial proliferation, germ cell differentiation, Sertoli cell steroidogenesis and protein secretion [3]. It is widely accepted that levels of several cytokines, such as IL-6, IL-8 and tumour necrosis factor α (TNF-α), increase in the presence of different pathologies [4–8] or seminal conditions in which sperm parameters are altered [9]. Even *Helicobacter pylori* chronic infection, caused by cytotoxin-associated gene A (CagA) positive strains, is concomitant with the rise in seminal plasma of inflammatory cytokine levels, which negatively affects sperm motility and determines sperm damage, reducing the reproductive potential of men [10].

The research has indeed demonstrated a strong association between inflammation in the male reproductive system and the consequent development of infertility [11]. Jiang et al. [12] reported that many cytokines have elevated concentrations in semen with high reactive oxygen species (ROS) levels, indicating a real relationship between cytokines and oxidative stress. At a physiological concentration, ROS are important to regulate sperm maturation, hyperactivation, capacitation, acrosome reaction and fertilisation. Spermatozoa are particularly vulnerable to lipid peroxidation (LPO) due to the high content of polyunsaturated fatty acids (PUFAs) in their plasma membrane. LPO produces lipid peroxides that accumulate in spermatozoa, creating a variety of decay end products, such as 4-hydroxynonenal, isoprostanes and malondialdehyde (MDA), which can be measured as indicators of oxidative stress [13]. MDA measurements, the most used method to detect LPO, can be performed either in seminal plasma [14–17] or sperm cells [9, 18]. It was demonstrated that several proinflammatory cytokines, either alone or in the presence of leukocytes, could cause LPO of the spermatozoa plasma membrane to such levels that can affect the sperm fertilising ability [3, 9, 19]. In particular, IL-33 is a member of the IL-1 cytokine family and can act as both cytokine and nuclear transcription factor. It is also classified as “alarmin” since its secretion upon necrotic cell death triggers robust proinflammatory responses [20].

Considering these studies, we performed semen analyses in 44 samples, evaluating the sperm parameters, including viability. IL-6 and IL-33 levels were then assessed in seminal plasma, as well as MDA concentrations in both seminal plasma and spermatozoa lysate. The relationships among these variables and semen parameters were statistically measured. We focused on the effect of seminal IL-6 and IL-33 on LPO measured in viable sperm cells. Finally, two small groups of patients with varicocele or positive semen cultures were extrapolated from the cases analysed and the variables compared with those of a group of fertile men.

## Materials and Methods

### Semen Samples and Study Design

Semen samples were obtained from 52 individuals (aged 24–37 years) who consecutively attended our Centre for semen analysis from January–June 2019. The 52 cases enrolled in this study fulfilled the following inclusion criteria: absence of systematic sperm defects, non-azoospermic men, and no history of radiotherapy, chemotherapy, chronic illness or medication, no use of drugs, alcohol or dietary supplements.

Hormone levels and bacteriological analyses were part of the clinical workup and assessed in a dedicated laboratory. The population studied showed normal concentrations of follicle-stimulating hormone (FSH), luteinizing hormone (LH) and testosterone (T). All patients provided bacteriological analyses of their semen samples and patients with positive semen cultures were identified as part of the group of “infection”.

All patients provided clinical and physical examinations and scrotal Eco-color Doppler, performed in a specialised medical clinic, to detect the possible presence of varicocele. Subclinical varicocele was not considered in this study.

For the comparison of variables, a group of 11 fertile men (aged 26–38 years), not affected by anatomical problems and/or infections and who fathered a child in the last three years, was considered.

The participants signed an informed written consent before participating in this research, accepting that their semen samples and the clinical data they supplied might be used for scientific purposes.

### Semen Analyses

The semen analyses of the patients enrolled in this study and fertile controls were performed following WHO guidelines [21]. We required subjects to abstain from sexual intercourse and masturbation for a period of 3–4 days before semen collection. Semen specimens were liquefied for 30 min at 37 °C; then semen volume, pH, sperm concentration and morphology were assessed according to WHO guidelines [21]. Sperm morphology was evaluated using the Papanicolaou test modified for spermatozoa [21].

Sperm viability was determined as follows: 10 μL of each semen sample and 10 μL of 0.5 % eosin Y (CI 45380) in 0.9% aqueous sodium chloride solution were mixed on a glass slide [21]. A few minutes after staining, the samples were examined by light microscope scoring red stained (dead) and unstained cells (alive). More than 300 sperm per sample were analysed. The possible presence of leukocytospermia (>1 × 10^6^ leukocytes/mL) was highlighted by peroxidase stain and those patients with leukocytospermia were excluded from this study.

After semen analyses, the samples were centrifuged at 400*g* for 15 min and the recovered seminal plasma was immediately examined under a microscope, ensuring the absence of cells, and the sample was then stored at −80 °C until use.

The spermatozoa were washed twice with phosphate buffer saline (PBS, pH = 7.4), centrifuged twice at 400*g* for 5 min to remove the residual seminal plasma and suspended in 1 mL of PBS. The sperm specimens were lysed through rapid freeze-thawing at −80 °C and + 35 °C, three times respectively. The samples were then centrifuged at 2500*g* for 5 min and the supernatant stored at −80 °C until analysis.

IL-6, IL-33 and MDA levels were assayed in seminal plasma, whereas MDA levels were measured in the spermatozoa of each sample.

### Seminal IL-6 and IL-33 Determinations

The determinations of seminal IL-6 and IL-33 were performed by enzyme-linked immunosorbent assay (ELISA, Human IL-6 BMS213/2CE BMS213/2TENCE Bender MedSystems GmbH, Vienna, Austria, and Human IL-33 ELISA kit, Besancon Cedex, France) and the results were expressed in pg/mL. For each sample, three specimens were determined and the calculation was carried out on the calibration curve, as reported in different kit manuals.

### MDA Determinations

The extent of LPO in lysed sperm cells and seminal plasma was estimated using MDA level calculation as follows: the same amount of tris-hydrogen chloride (HCl) 0.04 M and acetonitrile containing 0.1% butylated hydroxytoluene (BHT) was added to 0.5 mL of seminal plasma and 0.3 mL of lysed sperm cells. After pre-column derivatisation with 2,4-dinitrophenylhydrazine, the samples were extracted with 5 mL of pentane and dried under nitrogen. MDA-hydrazone was quantified by isocratic, reversed-phase high-performance liquid chromatography (HPLC) method with ultraviolet (UV) detection, as described by Shara et al. [22], with minor modification. For this purpose, a Waters 600 E System Controller HPLC (Milford, MA, USA), equipped with a Waters Dual λ 2487 UV detector (Milford, MA, USA) set at 307 nm, was used. A 5 μ ultrasphere ODS column C18 (Beckman, San Ramon, CA, USA) was employed to separate the hydrazone derivative at the flow rate of 0.8 mL/min with a mobile phase consisting of acetonitrile (45%) -HCl 0.01 N (55%). MDA concentrations were calculated by peak areas determined using an Agilent 3395 integrator (Agilent Technologies, Santa Clara, CA, USA). A calibration curve in the range of 0.2–10 nmol/mL was used to quantify the levels of MDA. Results were expressed as nmol/10^6^ cells and nmol/mL of MDA in sperm cells and seminal plasma, respectively.

Regarding the MDA levels directly evaluated in lysed spermatozoa, the results were adjusted considering the percentage of viable sperm assessed by the eosin Y test. This procedure enabled us to exclude from the calculation dead sperm with broken plasma membranes that had released their content in seminal plasma.

### Statistical Analyses

Statistical analyses were performed by IBM SPSS Statistics Software v.26. Because of the non-normal distributions (Kolmogorov-Smirnov test), data were reported as medians and interquartile range (IQR: 75°–25° centile). Spearman rho (*R*) coefficient was calculated to measure the correlation between variables. The Mann-Whitney *U* (MW) test was used to compare the variables of the two groups analysed, considering as cutoff the median value of IL-6. The significance of the differences among the fertile, infection and varicocele groups was evaluated with the Kruskal-Wallis test for independent groups. When a statistically significant difference was found among the groups, the Dunn’s post hoc test for multiple comparisons was applied between pairs of groups. A *p* value < 0.05 (two-tailed) was considered statistically significant.

## Results

We examined the semen samples of 52 patients; 10 out of 52 patients showed varicocele (II and III grade), four had a history of varicocele (ex varicocele), two individuals were characterised by treated cryptorchidism (ex cryptorchidism) and 13 patients showed positive semen cultures (infections). After sperm analysis, we excluded seven samples with leukocytospermia and one sample with a high percentage of germinal cells, to effectively examine MDA in human sperm cells and avoid contamination in the pellet. Lastly, the analysis of the semen samples of 44 patients were performed (eight with varicocele, three ex varicocele, two ex cryptorchidism, and nine with positive semen cultures: six patients’ semen cultures were positive for *Escherichia coli*, two for *Enterococcus faecalis* and one for *Ureaplasma urealyticum*). The characteristics are shown in Table 1. The median sperm concentration resulted between the 50th–75th centiles, median progressive motility percentage between the 5th–10th centiles, median percentage of sperm with normal morphology fell between the 10th–25th centiles and median percentage of sperm viability was between 25th–50th centiles [21].

**Table 1:** Characteristics of 44 semen samples

	Median	IQR
Volume (mL)	3.5	1.4
Sperm/mL × 10^6^	84.1	91.0
Progressive motility (%)	32.5	26.0
Normal forms (%)	8.0	11.0
Viability (%)	72.5	23.0
IL-6 (pg/mL)	21.5	27.2
MDA semen (nmol/mL)	1.15	3.73
MDA sperm (nmol/10^6^ cells)	0.05	0.06

*Note*: *IQR*, interquartile range

The median of IL-6 level was 21.5 pg/mL, whereas IL-33 was completely absent in all the semen samples analysed and not included in the tables and figures. The mean of MDA level in semen was 1.15 nmol/mL and in viable spermatozoa 0.05 nmol/10^6^ cells.

**Fig. 1:**
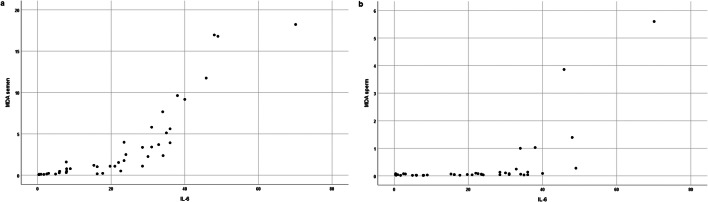
Panel (**a**) indicates scatter plot between IL-6 and MDA measured in the semen; panel (**b**) indicates scatter plot between seminal IL-6 and MDA measured in the spermatozoa

The correlations between variables are reported in Table 2. Significant positive correlations were observed among the semen parameters, such as sperm concentration, percentages of progressive motility, normal forms and viability. IL-6 level was positively correlated with MDA concentration, measured both in semen and spermatozoa (*p* < 0.01; Fig. 1) and negatively with sperm concentration, progressive motility, normal morphology and viability (*p* < 0.01). MDA levels measured both in seminal plasma and spermatozoa were negatively correlated with sperm parameters (*p* < 0.01). Finally, seminal MDA concentration showed a positive correlation with MDA level measured in viable spermatozoa.

**Table 2:** Bivariate correlation between all variables examined

	Volume (mL)	Sperm/mL 10^6^	Progressive motility (%)	Normal forms (%)	Viability (%)	IL-6 (pg/mL)	MDA semen (nmol/mL)	MDA sperm (nmol/10^6^ cells)
Volume (mL)	1.00	− 0.20	0.03	− 0.02	0.03	− 0.03	− 0.06	0.01
Sperm/mL ×10^6^		1.00	0.51**	0.44**	0.46**	− 0.54**	− 0.47**	− 0.53**
Progressive motility (%)			1.00	0.83**	0.96**	− 0.96**	− 0.96**	− 0.62**
Normal forms (%)				1.00	0.82**	− 0.77**	− 0.81**	− 0.44**
Viability (%)					1.00	− 0.92**	− 0.93**	− 0.57**
IL-6 (pg/mL)						1.00	0.94**	0.64**
MDA semen (nmol/mL)							1.00	0.94**
MDA sperm (nmol/10^6^ cells)								1.00

**p < 0.01

In semen, IL-6 and MDA levels showed an exponential relationship (Fig. 1a), whereas the trend between semen IL-6 levels and MDA concentration measured in viable sperm was different. In the latter case, MDA values were low and quite homogeneous until the IL-6 level reached a value of > 30 pg/mL; then, MDA level consistently increased (Fig. 1b).

To investigate the relationship between IL-6 and other variables, we clustered the data in two groups, considering the median value of IL-6 as the discriminant cutoff (IL-6 = 21.5 pg/mL). The median values of variables in the two groups are reported in Table 3.

**Table 3:** Descriptive analysis of all variables in groups with IL-6 *≤* 21.5 pg/mL and groups with IL-6 > 21.5 pg/mL

	IL-6 ≤ 21.5 (*n* = 22)		IL-6 > 21.5 (*n* = 22)		MW test
	Median	IQR	Median	IQR	*p* value
Volume (mL)	3.9	1.1	3.5	1.6	0.397
Sperm/mL ×10^6^	125.5	88.8	63.5	50.2	< 0.001
Progressive motility (%)	48	8	23	11	< 0.001
Normal forms (%)	12	10	5	4	< 0.001
Viability (%)	82	9	61	16	< 0.001
IL-6 (pg/mL)	6.9	14.0	33.4	11.1	< 0.001
MDA semen (nmol/mL)	0.26	0.74	3.95	6.94	< 0.001
MDA sperm (nmol/10^6^ cells)	0.03	0.03	0.09	0.40	< 0.001

*Note*: *MW test*, Mann-Whitney *U* test; *IQR*, interquartile range

The statistical comparisons showed significant differences (*p* < 0.001) among all variables, except for volume. In particular, we observed that semen quality was reduced, and the MDA concentrations measured in both semen and spermatozoa were significantly increased in the group characterised by IL-6 > 21.5 pg/mL.

Regarding the cases with varicocele, ex varicocele, ex cryptorchidism, and positive semen cultures defined as infection, we observed that six of eight individuals with varicocele, one of three ex varicocele men, the two ex cryptorchid patients and seven of nine patients with infection belonged to the group with IL-6 > 21.5 pg/mL. For this reason, we divided the population studied in two groups: (i) subjects with genitourinary infections (nine patients, group I) and (ii) subjects with varicocele (eight patients, group V). Then we included a group of fertile men (11 men, group F) for variable comparison (Table 4). The values of sperm concentration, progressive motility, viability and normal sperm morphology were significantly reduced in the infection and varicocele groups, compared to those observed in fertile controls (Table 4). On the contrary, the values of IL-6 level, seminal and sperm MDA concentrations were significantly increased in both infection and varicocele groups, compared to those detected in the control group (Table 4). Seminal IL-6 levels detected both in the infection and varicocele groups resulted in higher than the median of the distribution of the values in the 44 cases analysed (21.5 pg/mL), compared to the very low levels of IL-6 in fertile men.

**Table 4:** Median (IQR) of the variables studied in the three groups analysed and relative statistics

	Diagnosis			Statistics	
	Infection (*I*)	Varicocele (*V*)	Fertile men (*F*)	Kruskal-Wallis test *p* value	Dunn’s post hoc test *p* value
Volume (mL)	3.5 (1)	4.3 (2)	4.0 (1.5)	0.566	
Sperm/mL × 10^6^	76 (37)	73.5 (61)	152.0 (130)	< 0.01	*V* vs. *F* < 0.05 *I* vs. *F* < 0.05
Progressive motility (%)	22 (15)	26 (16)	50 (3)	< 0.001	*V* vs. *F* < 0.01 *I* vs. *F* < 0.001
Normal forms (%)	6 (3)	7 (5)	12 (10)	< 0.01	*V* vs. *F* < 0.01 *I* vs. *F* < 0.05
Viability (%)	56 (14)	66 (16)	83 (4)	< 0.001	*V* vs. *F* < 0.05 *I* vs. *F* < 0.001
IL-6 (pg/mL)	36 (9.5)	29.3 (12.9)	2.5 (5.5)	< 0.001	*V* vs. *F* < 0.01 *I* vs. *F* < 0.001
MDA semen (nmol/mL)	3.92 (7.97)	2.82 (3.75)	0.12 (0.22)	< 0.001	*V* vs. *F* < 0.05 *I* vs. *F* < 0.001
MDA sperm (nmol/10^6^ cells)	0.67 (0.87)	0.103 (0.126)	0.023 (0.012)	< 0.001	*V* vs. *F* < 0.01 *I* vs. *F* < 0.01

## Discussion

In the present study, we described the association between IL-6 levels evaluated in human seminal plasma and MDA measured in both semen and viable spermatozoa.

It is noteworthy that IL-33 was absent in all analysed semen samples, suggesting a role of a nuclear factor rather than secreted cytokine in human seminal plasma. Recently, Chatrabnous et al. [23] reported high levels of this cytokine in the blood serum of patients affected by prostate cancer. An interesting finding concerned the presence of IL-33 and its receptor ST2 in human follicular fluid [24]; however, further studies are necessary to explore the role of IL-33 in female and male reproductive systems.

Cytokines are part of the natural components of seminal plasma and are involved in normal male reproductive physiology. However, in many pathological conditions, their concentration increases and negatively influences sperm function [6]. For example, male accessory gland infection/inflammation (MAGI) and varicocele are conditions associated with oxidative stress [17, 25–27], and characterised by increased levels of cytokines [4–6], both in the presence and absence of leukocytospermia [28, 29]. MAGI are frequent, mostly chronic, pathologies affecting the sexual accessory glands. Generally, they remain asymptomatic and the identification of inflammation markers in seminal plasma is still an open challenge. IL-6, IL-8 [30] and other proteins can be considered the most promising markers for diagnosis and follow-up of MAGI [31]. In the group of patients studied, varicocele and positive semen cultures defined as infection were the most represented pathologies, but we cannot exclude the possible presence of asymptomatic MAGI. Unfortunately, we did not have information about bacterial inflammation of the male accessory glands, generally diagnosed by clinical, laboratory and ultrasound evaluation or by a specific questionnaire [32, 33].

The results of this research matched those reported in the literature concerning the negative relationship between semen cytokine concentrations and sperm parameters [5, 6, 34]. There is clear evidence that cytokines influence spermatozoa functions and can modulate pro-oxidant and antioxidant activities in the male genital tract [35]. For example, IL-1 α, IL-1 β and TNF-α are able to stimulate LPO by increasing ROS generation [3, 36] and seminal IL-6 showed positive correlations with membrane LPO [37, 38].

MDA, a marker of LPO, is widely used as a diagnostic tool to explore the damage that occurs to sperm cells exposed to oxidative stress [13, 39]. ROS concentrations are determined by the balance between the rates of ROS production and their quenching by antioxidant compounds that act as defense mechanisms. ROS can be generated by both exogenous sources, such as radiation and toxins, and endogenous sources, such as the effects of varicocele, the accumulation of damaged spermatozoa with an excess of residual cytoplasm, and the presence of immune cells [39]. In addition, the two major sources of ROS produced directly in sperm cells are the following: (i) nicotinamide adenine dinucleotide-phosphate (NADPH) oxidase system at the sperm plasma membrane level, and (ii) activity of the oxide reductase NADH-dependent in mitochondria that has been indicated as the main source of ROS in the sperm of infertile men [40]. Regarding the relationships between MDA levels measured in seminal plasma and sperm parameters, our results are consistent with the literature. In particular, sperm concentration [41, 42], motility [14, 16, 41–44], morphology and viability [15], showed significant negative correlations with MDA. Recently, Collodel et al. [17] demonstrated that seminal MDA is a marker of pathologies responsible for sperm motility reduction, such as urogenital infections and inflammatory status. However, in most studies, MDA is measured in seminal plasma and rarely in sperm lysate [9, 18]. Recently, Chyra-Jach et al. [9] reported that seminal MDA levels increase in males with sperm motility alterations and our findings are concordant with these results. In addition, we observed an exponential relationship between IL-6 level and MDA concentration measured in semen, indicating that this interleukin can be part of the oxidative stress pathway in human semen. By contrast, Chyra-Jach et al. [9] found that the level of MDA in spermatozoa lysate was reduced in patients affected by oligozoospermia, asthenozoospermia and oligoasthenozoospermia. The authors explained these paradoxical results with a possible mitochondrial dysfunction associated with decreased oxidative metabolism and possible leakage of MDA from the sperm cells damaged by LPO. This last point is very important to explain our different results. In the present study, the MDA values obtained from the sperm lysate were calculated considering the percentage of viable sperm assessed by eosin Y test [18]. This procedure allowed elimination from the calculation the dead sperm with broken plasma membranes that released their content in the seminal plasma. We observed significant negative correlations between MDA measured in spermatozoa lysate and motility, viability and a positive correlation with IL-6 measured in semen. This last relationship was interesting as the MDA values in viable spermatozoa were relatively low and homogeneous until IL-6 reached a concentration of approximately 30 pg/mL, after which the MDA amount started rising. It is interesting that in in vitro studies, Martinez et al. [3] considered as physiological an IL-6 concentration of 25 pg/mL, even though it is difficult to identify a physiological concentration of this cytokine in human semen since a number of patients examined in different studies are quite small and their characteristics different.

Despite the cellular MDA increase, the spermatozoa were still viable, and it is possible to hypothesise a positive reaction and protection of the antioxidant system [18]. The spermatozoa require the antioxidant-buffering ability of seminal plasma to counteract ROS, however they show a small amount of cytoplasm that contains intracellular enzymatic and non-enzymatic antioxidants [45, 46]. With this purpose, Chyra-Jach et al. [9] reported positive correlations between superoxide dismutase activity measured in spermatozoa lysate and sperm volume, sperm cell count, and rapid progressive motility.

Unfortunately, a limitation of our research lies in the fact that antioxidants were been measured, even if, in light of the data obtained, this field is worth exploring.

In addition, to verify the results achieved, we first clustered the data in two groups, considering the median of IL-6 concentrations (21.5 pg/mL) as the discriminant cutoff. In the group with low IL-6 levels, sperm parameters were significantly increased, whereas semen and sperm MDA were decreased. This methodology allowed us to highlight that most patients affected by varicocele, infections and men with a history of cryptorchidism belonged to the group characterised by IL-6 level > 21.5 pg/mL. For this reason, the variables of eight patients with varicocele, nine patients with infections and a new group of 11 fertile men were compared. The results confirmed that the high IL-6 concentration in the varicocele and infection groups was concomitant with the increased concentration of both seminal and spermatozoa MDA, indicating an association of this cytokine with LPO, as well as in cells. It is noteworthy that the medians of IL-6 concentration in the varicocele and infection groups were approximately 30 pg/mL. This value also represented the point on the scatter plot at which sperm MDA concentration started to rise as the level of IL-6 increased.

## Conclusions

We demonstrated that if IL-6 concentration increases in human seminal plasma, LPO increases in viable spermatozoa. The surge observed in LPO could be physiological when below a certain threshold, thus representing a pathological situation in living spermatozoa.

Obviously, the limit and meaning of LPO tolerance in living spermatozoa must be further explored. We are aware that more data and experiments are essential to confirm the results obtained in this study. It is also necessary to implement these data, considering the antioxidant capacity of spermatozoa and seminal plasma in relation to IL-6 level and other cytokines, particularly in groups of patients affected by different pathologies.

## Data Availability

The data generated and analysed during this study are included in this published article and are available from the corresponding author.
